# The governance of imported 2019-nCov infections: What can be learned from China’s experience?

**DOI:** 10.1186/s41256-022-00243-5

**Published:** 2022-03-09

**Authors:** Hao Li, Jiaxin He, Jiayu Chen, Shuning Pan, Jiehan Feng, Shuang Liu

**Affiliations:** 1 Global Health Institute/School of Public Health, Wuhan University, Wuhan, China; 2 School of Law, Henan University, Kaifeng, China; 3 Shenzhen Health Development Research and Data Management Center, Shenzhen, China; 4 Institute of International Law, Wuhan University, Wuhan, China; 5 Law School, Tianjin University, No.92, Weijin Road, Nankai District, Tianjin, 300072 China

**Keywords:** 2019-nCoV, COVID-19, Imported infection, Policy implication, China

## Abstract

Delta and Omicron variants of 2019-nCoV are still spreading globally, and many imported infections have been identified in China as well. In order to control the spread chain from imported to local, China has implemented the dynamic Covid-zero policy. In this article we summarized China’s governance models and practices of fighting potential imported infections in two directions. One targets at international travelers, which can be outlined as four lines of defense: customs epidemic prevention, quarantine upon arrival, relevant laws and regulations, and community tracking. The other is against other vectors potentially carrying 2019-nCoV, which can be outlined by three lines of defense: customs epidemic prevention, disinfection and personal protection, and information management. However, there are still some challenges that are yet to be addressed, such as illegal immigration, accidental occupational exposure to 2019-nCoV, etc. China’s experience indicates that no country can stay safe during the global pandemic as long as there are local outbreaks in other countries, and active prevention and control measures based on science and a complete set of laws and regulations are still necessary at current stage. What’s more, accountable government commitment and leadership, strengthened health and social governance systems, and whole society participation are required. It is suggested that the global community continue to closely cooperate together and take active rather than passive actions to block the potential imported 2019-nCoV from causing local spreading.

## Background

The 2019-nCoV has been mutating over time, and variants such as Delta and Omicron are spreading more easily between people [1]. The Delta variant of coronavirus disease 2019 (COVID-19) was first detected in India in October 2020 [2]. The Omicron variant, which is more contagious than the Delta variant, was reported by South Africa to the World Health Organization (WHO) in November 2021 [3]. According to specimens collected worldwide from 12 December 2021 to 10 January 2022, 41.4% of them were Delta and 58.5% were Omicron [4].

China is also confronted with the risks of the imported 2019-nCoV. On 13 December 2021, an international traveler to Tianjin metropolis was found asymptomatically carrying the Omicron variant [5]. The next day, Guangzhou city reported its first imported case of Omicron [6]. Thanks to the closed-loop management of international travelers and upgraded quarantine policies in China, the two persons did not trigger widespread local transmission. In fact, local transmission caused by imported infections have occasionally occurred in China (Table 1) in the past two years [7]. As a continuous response, China has developed a series of measures and policies to prevent and control imported infections and subsequent local transmissions.

**Table 1:** Some examples of local transmission caused by imported infections in China

First date of confirmed cases	First location of confirmed cases	Possible causes
11 June 2020	Beijing	By an environment-to-human transmission from contaminated food imported via cold-chain logistics
22 July 2020	Dalian	Clustered outbreak due to exposure at a processing facility of frozen seafood products
24 October 2020	Kashi	Transmission from offshore containers
8 November 2020	Tianjin	Contact with imported frozen pork with positive nucleic acid test (NAT)
9 November 2020	Shanghai	Exposure to an inbound aviation container
7 December 2020	Chengdu	Contact with waste in a quarantine facility of confirmed cases of international travelers
25 December 2020	Beijing	Contact with a confirmed asymptomatic case of international traveler
21 May 2021	Shenzhen	Exposure to international cargo ships
20 July 2021	Nanjing	Exposure to an inbound flight

Data source: The official websites of China’s provincial and municipal health commissions or press conferences on epidemic prevention and control

At first, China’s strict dynamic Covid-zero policy against imported infections and local transmission based on science [8] have not been fully recognized by other countries. With the deterioration of the global spread of the Delta and Omicron variants, however, many countries started to recognize the importance of general prevention and control policies instead of merely depending on vaccination and herd immunity. Although some types of vaccines have been developed and released to the market, most of them have been ordered and purchased by high-income countries (HICs), while many low- and middle- income countries (LMICs) have very limited access to them [9], making herd immunity approach difficult to achieve. Despite the fact that vaccination can significantly reduce fatality, the development of new vaccines always lags behind virus mutations, which may reduce vaccine effectiveness [10]. Therefore, in addition to accessible, affordable and effective vaccination for the general public, comprehensive monitoring and governance of imported cases always remain important in daily actions. As recommended by the WHO, Public Health and Social Measures (PHSM) shall be widely adopted globally in the current pandemic response [11].

In this article, we aim to summarize China’s governance experience of blocking imported infections from causing local transmissions and provide policy implications for other countries. Two governance models are discussed. One for international travelers, and the other for other vectors. 

## China’s governance model against imported infections from international travelers

**Fig. 1:**
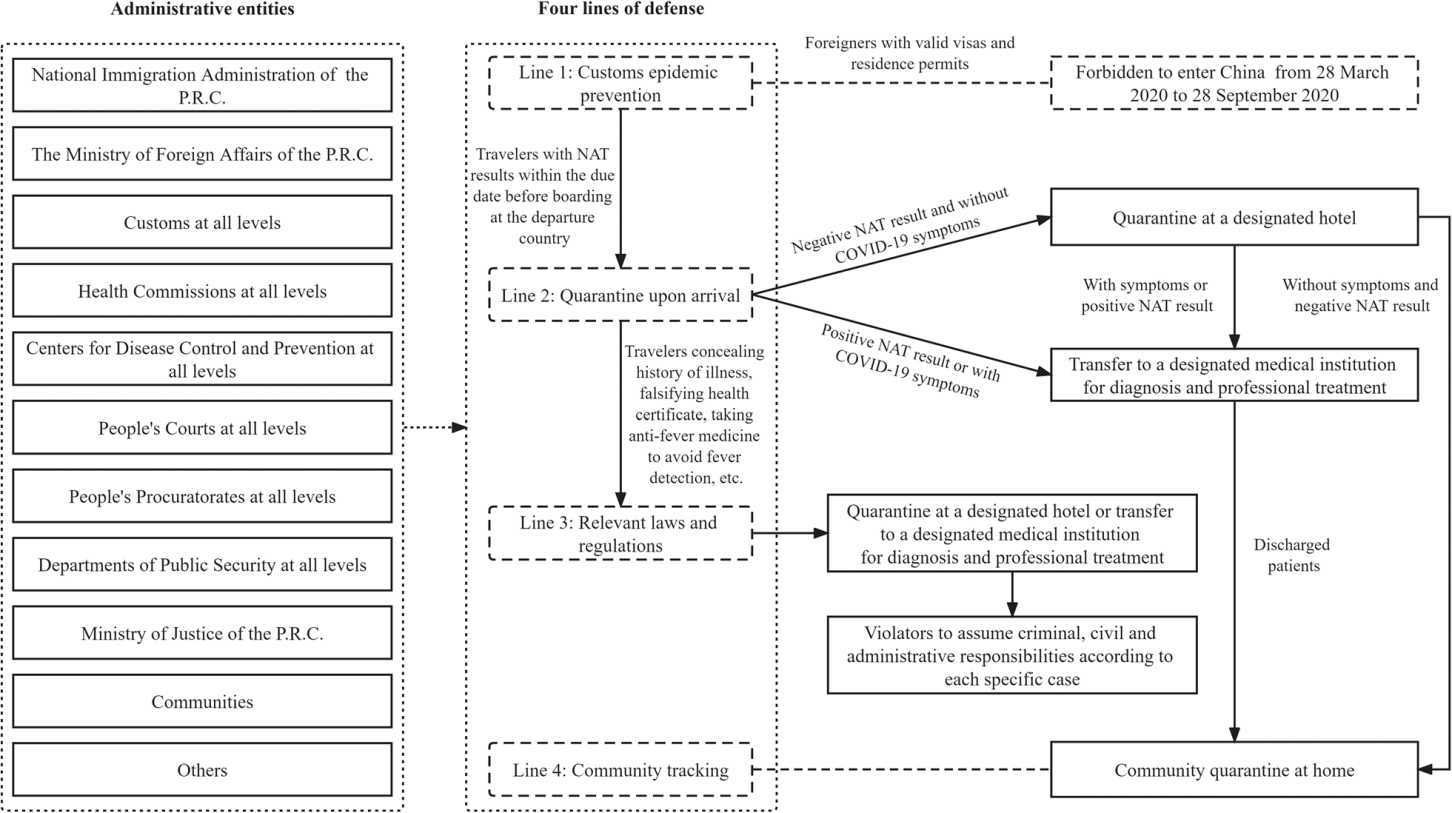
China’s governance model against imported infections from international travelers

Figure 1 depicts China’s governance model against imported infections from international travelers (Model 1), which was compiled from a review of literature and existing practices [12–18]. It can be seen that administrative entities atall levels can all play their respective roles in the governance of international travelers led by the Joint Prevention and Control Mechanism of the State Council of China (JPCMSCC). These entities coordinate with each other based on four lines of defense against international travelers issued by the JPCMSCC, which consist of customs epidemic prevention, quarantine upon arrival, legal defense, and community tracking. The four lines of defense have been implemented by the Chinese government at all levels strictly based on relevant laws and regulations such as the *Frontier Health and Quarantine Law of the People's Republic of China* and the *Law of the People's Republic of China on Prevention and Treatment of Infectious Diseases* in parallel with the *International Health Regulations (2005)* released by the WHO. The four lines of defense are summarized as follows:

- **Line 1: Customs epidemic prevention**. Since 28 March 2020, foreigners with valid visas and residence permits had been denied to enter China [19], and Chinese citizens abroad could only return through chartered flights arranged by the Chinese government. Starting from 28 September 2020, foreigners are re-permitted to enter China, with the requirement to submit and provide negative nucleic acid testing (NAT) results by the due date before boarding at the departure country [20, 21]. The NAT result qualification is also applicable to Chinese passengers. All international passengers are required to provide both their health code status and health status statements for pass qualification. Passengers failing to meet the requirements will be denied to board.
- **Line 2: Quarantine upon arrival**. On 1 April 2020, the Chinese government stipulated that anyone who would enter China shall take NAT at the point of entry [22]. If a passenger’s test result is negative and he/she shows no COVID-19 symptoms, he/she will be allowed to pass the Customs. He/she shall be then quarantined at a designated hotel for a minimum of14 days, and the exact quarantine periods vary by the stipulations of destination cities. Guangzhou government, for example, released the policy of “14 days of centralized quarantine at hotels + 7 days of community quarantine at home + 7 days of self-health monitoring” on 16 November 2021 [23]. During the quarantine, he/she shall take NATs regularly. If the passenger’shis/her test result is positive, he/she will be transferred directly to a designated medical institution for diagnosis and professional treatment. If his/her test result is negative and he/she shows no COVID-19 symptoms after 14 days of centralized quarantine, he/she will be allowed to go to their destination community and stay for another 7 or 14 days of home quarantine.
- **Line 3: Relevant laws and regulations**. According to relevant laws and regulations in China, violators will assume criminal, civil, and/or administrative responsibilities depending on specific cases such as concealing history of illness, falsifying health certificate, taking anti-fever medicine to avoid fever detection, etc. [24]. In order to constrain the spread of 2019-nCoV from customs, on 16 March 2020, *the Opinions on Further Strengthening Frontier Health and Quarantine Efforts and Lawfully Punishing Illegal and Criminal Activities that Impair Frontier Health and Quarantine Regulation* were jointly released by the Supreme People's Court, the Supreme People's Procuratorate, the Ministry of Public Security, the Ministry of Justice, and the General Administration of Customs [25]. The judicial interpretation issued by the above five central authorities requires passengers to cooperate with customs and other relevant administrative entities. Anyone violating the laws and regulations will be punished in order to maintain social order and prevent local transmission [26].
- **Line 4: Community tracking**. There is a complete governance system in the community [27] to carry out mobility management, screening of returning residents, and monitoring the health status of returning residents. In general, after 14 days of centralized quarantine at a designated hotel, an additional 7 to 14 days of community quarantine at home shall be required. Specific quarantine policies at the local community level are subject to change, depending on the developing conditions of the epidemic in local settings.

China’s governance model against imported infections from international travelers has significantly lowered the number of imported cases. However, in practice, some unusualsituations need to be addressed separately. For example, illegal immigration can result in local transmissions, especially given the long borderline that is hard to monitor. On 13 September 2020, Ruili, a border town next to Myanmar, reported 2 new imported cases [28]. Both cases had smuggled into China from Myanmar and escaped the procedures of the first two lines of defense [29]. Such unexpected traveler mobility had caused a huge panic among Ruili People. Moreover, border trade, transnational marriage between China and Myanmar, difficulty of border control, persistent illegal immigration, and the long-lasting fight against local outbreaks caused by occasional imported cases have all generated huge pressure to Ruili. In response, the Ruili government has taken strict measurements to prevent local outbreaks by strengthening border defense lines, strictly registering people traveling across the border, speeding up the third dose of vaccination, and conducting mass NATs regularly.

## China’s governance model against imported infections from other vectors

**Fig. 2:**
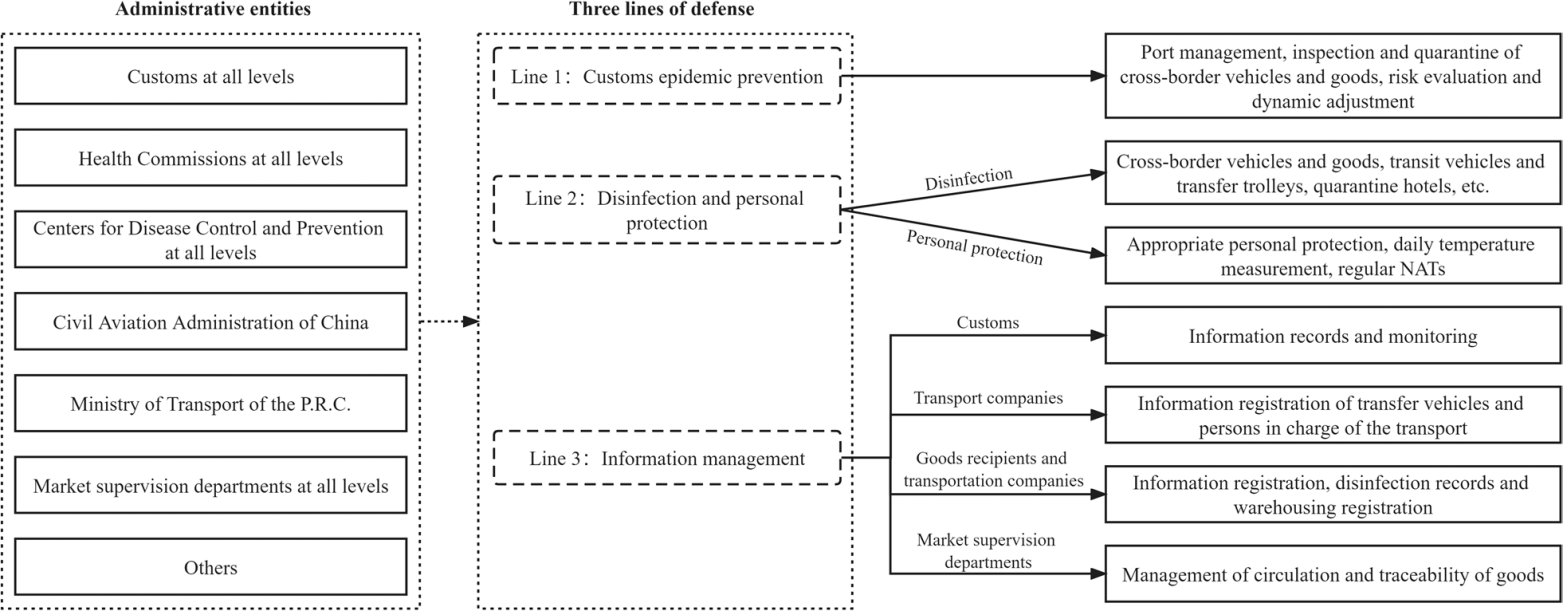
China’s governance model against imported infections from other vectors

In theory, it is necessary to focus on the governance of not only imported infections from human, but also other vectors such as inbound transportation vehicles, goods, etc. Current evidences indicate that international containers and imported cold chain products can carry coronavirus [30–32], and possibly cause human contact infections. Such transmission risks call for the need to extend our focus on not only human-human transmission, but also possible transmission from the environment. Based on existing literature and practices in China [33–36], three lines of defense against imported infections from other vectors can be depicted in Fig. 2 (Model 2), which mainly target at coronavirus that may enter the country through goods, air flights, road and water vehicles, and other carriers. The three lines of defense are summarized as follows:

- **Line 1: Customs epidemic prevention**. Land, air and sea ports shall be shut down partially or entirely if required according to a given set of standards. Inspection and quarantine of cross-border vehicles and goods shall be strictly implemented. At the same time, the customs shall closely track the trend of the global pandemic, carry out rapid risk assessments, and dynamically adjust the application of prevention and control measures at the ports based on information updatesfrom the source countries and regions.
- **Line 2: Disinfection and personal protection**. Inbound cross-border vehicles and goods, transit vehicles and transfer trolleys, and quarantine hotels shall be thoroughly disinfected. For inspection and quarantine staff at ports and inspection sites, drivers and logistical personnel of import goods, and other relevant groups, it is required for them to perform appropriate personal protection, measure daily body temperature, and take regular NATs.
- **Line 3: Information management**. Information of inbound vehicles, passengers, goods, and relevant personnel shall be recorded and monitored throughout the whole defense procedure. Transport companies shall register the information of their transport vehicles and persons in charge of the transport. The recipients of imported goods and the transportation companies shall cooperate with the customson information registration, disinfection records, and warehousing registration. Market supervision departments shall manage the circulation of goods and archive their traceability.

The establishment and application of Model 2 have reduced potentiallocal outbreaks caused by 2019-nCoV. For example, on 21 December 2021, during the routine monitoring of a batch of banana imports from Cambodia, the Centers for Disease Control and Prevention in Chifeng City of China, detected two samples (one from bananas and the other from the outer packaging of the bananas) with positive NAT results [37]. Fortunately, the bananas had not been sold yet, and were sealed and disposed of instead. People who had direct contact with the goods all followed appropriate control measures, and relevant places and vehicles were disinfected as well. The detection of infected goods and the ensuring proper actions prevented the potential spread of 2019-nCoV.

However, the application of Model 2 may not fully block imported infections caused by other vectors. For example, on 20 July 2021, seven locally transmitted cases and two asymptomatic cases were identified through weekly screening of target groups at Nanjing Lukou International Airport [38]. In the following two weeks, the epidemic in Nanjing had spread to some provinces in China. Preliminary evidence suggested that international flight cleaners who were exposed to a contaminated environment during cabin cleaning led to human-to-human transmission [39], indicating the necessity to segregate the staff of domestic and international flights. Therefore, neglect by relevant staff, lack of terminal disinfection, viability of the virus in the environment, together with imported goods and vehicles that may carry the coronavirus, had all increased the pressure on the prevention and control of imported infections.

## Governance and policy implications for other countries

China’s prevention and control experience indicates that, even through China has brought local outbreaks under control, it can still suffer at any time from imported infections by international travelers and other vectors, which can rapidly ignite a new local outbreak. With sufficient health promotion in place by the whole society, people are encouraged and more willing to get vaccinated, continue to wear masks when going out, and comply with other prevention and control measures [40]. Regular surveillance are still applied when moving from one city to another. All these measures implemented with solidarity from the whole society [41], have made China a safe country to live and work, and laid the foundation for supporting economic growth and contributing to the global fight against the pandemic. However, no country can stay safe as long as there are local outbreaks in other countries. If no measures are taken to prevent imported infections, when a new fatal variant (like Delta) emerges, the society may once again fall into chaos with a huge number of infections and deaths. In the long run, the virus may gradually mutate and reduce its virulence until it can co-exist with human-being. However, it is still unclear how long this may take. From this aspect, active prevention and control measures are more preferable than passive responses. This is particularly important to not only residents in LMICs but also disadvantaged and vulnerable populations in HICs.

China’s experience has served as an example that shows taking active actions can help reduce the risks of imported infections from other countries. For example, a large number of people have been infected with the Delta variant globally, while by 12 January 2021, only 202 cases were confirmed in China [42]. Even though the imported Delta variant caused a local outbreak in Xi’an city in December 2021, the situation was brought under control in about one month [43]. The 2022 Beijing Winter Olympics offers another example of applying China’s governance models to prevent and control infected infections [44]. All these practices and evidences indicate that China’s active governance models are effective for protecting the lives of the people. By forming a joint prevention and control mechanism, countries can benefit from China’s best practices among others to develop their own guidelines and governance models. However, the following aspects should be paid special attention to for more in-depth reforms of the governance system:

1. Accountable government commitment and leadership. In the governance of imported infections, political commitment is key to the successful implementation of prevention and control. Governments at all levels must take the lead and closely work together to fight the epidemic. In China, in many cases government officials were dismissed from their positions for their neglect of duty in the fight against the outbreak [45, 46]. In China, the government's commitment to “Putting people’s lives first” has been widely recognized by the people [47]. Therefore, public trust and solidarity have been formed and the overwhelming majority are willing to comply with the guidelines.
2. Strengthened health and social governance systems. It is necessary to consider whether the existing health system and health service capacity can support large-scale testing and quarantine, clinic treatments, and dynamic Covid-zero policy actions. Furthermore, complete and effective social governance systems should be in place as well in supporting the functioning of the health system. For example, logistical support system needs to meet the basic needs of not only medical professionals and patients, but also international travelers and staff in quarantine. Imported goods and relevant facilities of logistics companies need to undergo a series of procedures for disinfection. The infrastructure of information communications technology shall be capable of supporting the monitoring of human mobility and other vectors [48]. Information and data management should be arranged for accurate collection and analysis of epidemic-related data, effective screening of erroneous/false information, open sharing of health research data, and user information behaviors [49]. Besides, the legal system shall allow the use of patients’ health status and mobility data for the fight against the epidemic.
3. Whole society participation. In China, the whole society has been convinced that, if an outbreak is not under control, health resources will not be sufficient to meet the needs for quarantine and treatment, causing numerous lives to be lost. Thus, to protect their families and continue to work for a better life, people are willing to sacrifice their personal interests for collective interests. The overwhelming majority of Chinese citizens have been vaccinated [50] and abide by the PHSM in their daily life. In addition, these rules are also applied to international travelers to fight potential imported cases.

## Conclusion

China puts people and their lives first when fighting the 2019-nCoV, and takes the initiative to fight the epidemic with dynamic Covid-zero policy actions based on science and a complete set of laws and regulations. These policy actions have thus saved countless lives. Based on China's experience, other countries are urged to fight local outbreaks on the one hand, and collaborate with the global community with solidarity to fight both imported and exported infections on the other. Each country should also encourage their people to abide by the PHSM, and actively implement the requirements stipulated in the International Health Regulations (2005), so as to integrate them into their own health systems. Each country should support the WHO to establish a legal framework of convention on pandemic preparedness and response, and transform their political commitment into sustained and effective actions. With the pandemic yet to be brought under control, the global community, including governmental and nongovernmental international organizations, still need to endeavour to carry out health promotions, invest more resources in the research and development of vaccines and medicines, improve the supply, accessibility and affordability of vaccines and medicines, and increase vaccination coverage until the global fight is over.

## Data Availability

Not applicable.

## Abbreviations

COVID-19: Coronavirus disease 2019
HIC: High income countries
JPCMSCC: The Joint Prevention and Control Mechanism of the State Council of China
LMIC: Low- and Middle Income Countries
NAT: Nucleic acid testing
PHSM: Public Health and Social Measures
WHO: World Health Organization
